# Measurement of Antioxidant Capacity of Meat and Meat Products: Methods and Applications

**DOI:** 10.3390/molecules26133880

**Published:** 2021-06-25

**Authors:** Noemí Echegaray, Mirian Pateiro, Paulo E. S. Munekata, José M. Lorenzo, Zakariya Chabani, Mohamed A. Farag, Rubén Domínguez

**Affiliations:** 1 Centro Tecnológico de la Carne de Galicia, Rúa Galicia Nº 4, Parque Tecnológico de Galicia, San Cibrao das Viñas, 32900 Ourense, Spain; noemiechegaray@ceteca.net (N.E.); mirianpateiro@ceteca.net (M.P.); paulosichetti@ceteca.net (P.E.S.M.); jmlorenzo@ceteca.net (J.M.L.); 2 Área de Tecnología de los Alimentos, Facultad de Ciencias de Ourense, Universidad de Vigo, 32004 Ourense, Spain; 3 International Business Department, Management Faculty, Canadian University Dubai, Dubai 117781, United Arab Emirates; zakariya.chabani@cud.ac.ae; 4 Department of Biology, School of Sciences and Engineering, The American University in Cairo, New Cairo 11835, Egypt; Mohamed.Farag@pharma.cu.edu.eg; 5 Pharmacognosy Department, College of Pharmacy, Cairo University, Kasr El–Aini St., Cairo P.B. 11562, Egypt

**Keywords:** antioxidant measurement methods, free radicals, oxidation, meat industry, ABTS, DPPH, FRAP, ORAC

## Abstract

At present, a wide variety of analytical methods is available to measure antioxidant capacity. However, this great diversity is not reflected in the analysis of meat and meat products, as there are a limited number of studies on determining this parameter in this complex food matrix. Despite this, and due to the interest in antioxidants that prevent oxidation reactions, the identification of antioxidants in meat and meat products is of special importance to the meat industry. For this reason, this review compiled the main antioxidant capacity assays employed in meat and meat products, to date, describing their foundations, and showing both their advantages and limitations. This review also looked at the different applications of antioxidant properties in meat and meat products. In this sense, the suitability of using these methodologies has been demonstrated in different investigations related to these foods.

## 1. Introduction

Meat and meat products are susceptible to spoilage due to their rich nutritional compositions [1]. Specifically, the first non–microbial cause of deterioration in the quality of these foods is the oxidation process, particularly the oxidation of lipids with a high content of polyunsaturated fatty acids [2,3]. Furthermore, although to a lesser extent, protein and pigment oxidation in meat and meat products also take place during the deterioration of these foodstuffs [4]. However, the susceptibility of meat to oxidation differs, depending on animal species [5], with beef being considered one of the most susceptible to oxidation processes [6]. Moreover, the diet supplied to the animals, the animal breed, the muscle type, and anatomical location has also been found to affect meat oxidation.

Generally, the first changes observed in the oxidation processes have to do with the sensory quality of the meat, including changes in color, texture, and the appearance of off–flavors and off–odors [3,7,8,9]. Specifically, the oxidation of meat causes loss of color (due to the oxidation of heme pigments) [10], damages texture attributes (because oxidative processes reduce protein solubility and water retention capacity) [11], and causes undesirable rancid odors generated by the formation of compounds derived from lipid oxidation with low detection threshold values, such as aldehydes and ketones) [12]. These modifications have a direct influence on consumer acceptance [13,14]. In this way, the shelf life of meat and meat products is determined by the moment in which the consumer detects the modifications produced by oxidative processes [15]. As a consequence, the actions that favor oxidation in the meat industry must be controlled in order to minimize economic losses at the industrial level [7,16]. Moreover, oxidative processes lead to a nutritional loss of meat because the reactions involved generate a decrease in essential fatty acids and essential amino acids, causing a loss of antioxidant vitamins [3]. Furthermore, the oxidation of meat has toxicological implications because different compounds with toxic properties and free radicals are generated during the oxidative processes [17,18,19]. In this regard, lipid oxidation originates many primary and secondary by–products, such as cholesterol oxides, malonaldehyde, and 4–hydroxynonenal, which are known as carcinogenic potentials [20]. Furthermore, it is known that carbonyl compounds and hydroperoxides (derived from protein and lipid oxidation, respectively) can affect cellular signal transduction and damage DNA [10]. In addition, free radicals generated during oxidative processes can increase oxidative stress in the human body [21]. Concretely, it has been seen that excessive amounts of reactive oxygen species are directly or indirectly involved in diverse human diseases, such as cancer, inflammatory sickness, diabetes, autism, Alzheimer’s disease, Parkinson’s sickness, atherosclerosis, heart failure, fatty liver, chronic fatigue syndrome, obesity, and depression [10]. In the course of oxidation, a series of complex reactions take place, which are favored by the action of reactive oxygen (ROS), nitrogen (RNS), and sulfur (RSS) species [4]. Specifically, ROS, such as the hydroxyl radical (OH^•^), peroxyl radical (ROO^•^), hydroperoxyl radical (HO_2_^•^), and alkoxy radical (RO^•^) are the main radicals that favor the triggering of oxidation [3,22]. These free radicals are highly unstable and active, their main targets being lipids, proteins, and pigments, thus initiating the oxidation pathway [4]. In the case of lipid oxidation, the reactive species originate a series of chain reactions that generate hydroperoxides, which rapidly decompose, causing a large number of secondary compounds that include hydrocarbons, aldehydes, ketones, alcohols, esters, and acids [3]. Similarly, proteins are easily oxidized by the action of free radicals since these reactive substances produce the cleavage of peptides, favoring the proteolysis process [23], and the formation of carbonyl compounds, such as α–aminoadipic and γ–glutamic semialdehydes [24]. Finally, free radicals can also mediate the oxidation of meat pigments as they transform deoxymyoglobin (an unstable form of myoglobin pigment) into the oxidized form metmyoglobin, damaging the color of the meat and meat products [25,26]. On the other hand, oxidation reactions are affected by various intrinsic factors, such as the presence of other pro–oxidants, different than free radicals (such as metals and pro–oxidant enzymes) or the presence of antioxidants (such as vitamins, certain enzymes, and peptides), which determine the oxidative stability of the meat by favor, or decrease oxidation reactions, respectively [3,17]. On this matter, the increase in antioxidant compounds versus pro–oxidant substances in meat and meat products play a special role in enlarging the shelf life of these foodstuffs [4,27]. Specifically, the principal approach by the meat industry to decrease oxidation processes is the enrichment of antioxidants in meat and meat products [3,28,29]. Nonetheless, due to the current trends of consumers who reject the use of synthetic antioxidants [30,31,32], on account of their association with risks to human health, and their possible carcinogenic effects [33,34], the food industry has opted for different strategies to increase the antioxidant substances of meat and meat products in order to improve their oxidative stability [16]. Thus, in fresh meat, the possible enhancement of oxidative status has been studied through the use of different natural diets in animal husbandry [35,36,37], or via the use of different native breeds [38,39], meanwhile, in meat products, there have been attempts to improve this parameter by the addition of different natural antioxidants [40,41,42,43,44].

This current tendency has made it necessary to use techniques to determine the antioxidant status in meat and meat products, in addition to the traditional determination of oxidation level. In this sense, there is a special interest in tests that determine antioxidant capacity. Initially, the concept of antioxidant capacity originated in the field of chemistry and was later adapted to other scientific areas, such as biology, epidemiology, and nutrition [45]. Therefore, the analysis of antioxidant capacity was also incorporated into the determinations of certain food products, with the aim of measuring and investigating the antioxidant property and capacity of frequently consumed nourishments [46]. However, many of these studies have focused on vegetables, fruits, and spices [47], while in meat and meat products, there are limited investigations that evaluate antioxidant capacity [48]. Despite this, various techniques have been shown effective in determining the antioxidant capacity of meat and meat–based products [49]. Therefore, the main objective of this manuscript was to perform a review of the most employed techniques for determining the antioxidant capacity in meat and meat products. Thus, both the fundamentals of the main techniques and their applications to meat and meat products have been described, providing certain considerations that could help promote their incorporation in routine meat analysis.

## 2. Determination of Antioxidant Capacity in Meat and Meat Products

### 2.1. Extraction of Antioxidant Compounds from Meat and Meat Products

The extraction of the compounds that exert the antioxidant capacity is a crucial step in determining the antioxidant capacity of any food [50,51]. With meat as no exception, the extraction processes acquires special interest for the subsequent correct analysis. In this regard, solid–liquid extraction is most frequently used in meat matrices. However, this process is performed in different ways, because various conditions can be employed for this purpose (for instance, distinct solvents, times, and temperatures of extraction) [47]. 

The first extraction differences are found in the starting sample, which can be used fresh [27,35,38] or lyophilized to favor the concentration of antioxidant compounds [14,42,47]. Moreover, the extracting solvent to be utilized also differs according to the work. For example, Perna et al. [52] and Simonetti et al. [38] used 0.05 M phosphate buffer to extract the antioxidant compounds, while other authors utilized 80% methanol [35], 100% methanol [14,42], water and chloroform [27], 0.01 N hydrochloric acid [39], and 100% ethanol [41,53] in meat or its derivatives. Additionally, the operations followed in the extraction of antioxidant compounds also differ according to the research consulted. Nevertheless, in general, the most employed actions involve homogenizing the sample with the solvent selected and subsequent centrifugation and filtration of the supernatant obtained, which will be the liquid extract to be analyzed, using the different antioxidant capacity techniques. In addition to these operations, the use of an ultrasound has been utilized in the management of antioxidant extracts from meat [35,38,52], since it favors the release of antioxidants from the cell.

As described above, it can be deduced that the extraction of antioxidant compounds in meat and their derivatives present a large source of variations between works, and in some cases, they are not reliable. Even due to the distribution of antioxidant compounds in the meat matrix, some of these substances may remain unextracted, because the methodology, based on the solid–liquid extraction, only takes into account the soluble and removable fraction, underestimating the antioxidant capacity of the food [54]. To avoid these drawbacks, Gökmen et al. [55] developed a technique that permits the direct measurement of antioxidant capacity in solid samples without prior extraction, eluding all hydrolysis and solvent extraction steps. Specifically, they proposed the direct measurement of lyophilized samples. That is, they accomplish the antioxidant capacity tests directly with the freeze–dried food. Thus, Gökmen et al. [55] developed the QUENCHER (acronym of QUick, Easy, New, CHEap, and Reproducible) method. Nevertheless, despite the advantages offered by this direct trial, few studies have determined the antioxidant capacity through the QUENCHER technique [54,56], which makes it difficult to compare the antioxidant capacity results from different research. 

### 2.2. Antioxidant Capacity Assays Frequently Employed in Meat and Meat Products

The measurement of antioxidant capacity permits determining the ability of certain molecules to eliminate free radicals or to transfer an electron to reduce an oxidant [57]. However, there is no single and reliable method that covers all aspects of the study of these properties, but there are currently different procedures [37,58] that can be employed for the broad analysis of meat and meat products. In fact, antioxidant capacity should not be based on a single antioxidant test model, but must be checked on the study of different determinations, which allows the detection of different target substances [59]. Generally, the methods for determining antioxidant properties of meat components can be divided according to the chemical reactions involved into hydrogen atom transfer (HAT)–based, electron transfer (ET)–based, and mixed mode (HAT– and ET–based) techniques [58,60] (Table 1).

### 2.3. HAT–Based Methods

**Figure 1:**
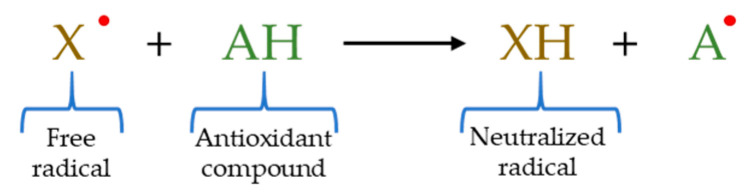
Reaction mechanism of HAT–based methods.

HAT–based assays are characterized by detecting the ability of an antioxidant to extinguish free radicals by donating hydrogen (Figure 1) [61]. Concretely, in these methods the oxidant reacts with goal compounds, called probes, creating changes in their spectroscopic characteristics (absorbance, fluorescence, and luminescence) where antioxidants compete against the probe for the associated oxidant [48]. HAT-based techniques include oxygen radical absorbance capacity (ORAC) assay; hydroxyl radical averting capacity (HORAC) technique; total peroxyl radical trapping antioxidant parameter (TRAP) assay; low–density lipoprotein (LDL) oxidation method; total radical scavenging capacity assay (TOSCA); β–carotene bleaching assays; and chemiluminescent assay [58]. Nevertheless, despite the great variety of HAT–based techniques, the use of these methods in meat and meat products has been very limited to date, highlighting only the ORAC and HORAC assays as techniques utilized to determine the antioxidant capacity in this type of nourishment.

#### 2.3.1. Oxygen Radical Absorbance Capacity (ORAC) Assay

The ORAC assay is based on the work reported by DeLange et al. [62]. This technique is a revolutionary new test–tube determination that can be employed to screen the antioxidant power of foods [58], including meat [40,47]. This methodology is supported by the measurement of the scavenging capacity against peroxyl radicals (ROO^•^), reflecting the classical radical chain breaking antioxidant capacity by hydrogen atom transfer [63]. Concretely, a peroxyl radical generator compound and a fluorescent substance are employed in this determination, which are usually 2,2′–azobis(2–amidinopropane) dihydrochloride (AAPH) and 3′,6′–dihydroxy–spiro[isobenzofuran–1[3H],9′[9H]–xanthen]–3–one (fluorescein) or 2′,7′–dichlorodihydrofluorescein diacetate (dichlorofluorescein), respectively [61]. Thereby, the thermal decomposition of AAPH in an aqueous buffer provides a constant flow of ROO^•^ while fluorescein or dichlorofluorescein acts as the oxidizable target, being the molecular probe that monitor the progress of the reaction via the emitted fluorescence [64]. In this way, the measurement of the decrease rate in fluorescence in the presence of ROO^•^ over time is performed, the excitation and emission wavelengths being 485 and 520 nm, respectively [47]. At the same time, the quantification of the amount of a non–fluorescent compound is produced since the fluorescent probe is transformed into a non–fluorescent product when reacting with ROO^•^ [59]. This process is normally accomplish in a microplate reader equipped with a fluorometer over short time intervals (around 1 min) for extended periods (greater than 30 min) [65]. Thus, in the presence of antioxidant substances, the decay of fluorescence is inhibited and the antioxidant capacity can be calculated [66]. Expressly, the quantification of ORAC values are normally reported as trolox equivalents since the trolox is usually used as a standard [61], and are based in the area under the curve that represents the oxidation of the probe along time [67].

One of the main advantages of the ORAC test is that it is particularly useful for samples that often contain multiple ingredients and have complex reaction kinetics, such as meat, because permits the detection of antioxidants that exhibit different lag stages [64] by representing the lag time, the initial rate, and the total inhibition in a unique value [61]. Furthermore, the ROO^•^ free radicals generated in this assay are found naturally in biological systems, so they can be representative of food systems. On the other hand, although the ORAC assay is a technique that initially only allows the detection of hydrophilic antioxidant compounds, it can also be adapted for the detection of lipophilic antioxidants [68]. However, this adjustment may necessitate the modification of the free radical generation source, the fluorescence emitting substance and/or the solvent usually employed, which may decrease the efficiency of the method. Despite this, it has been seen that the use of 2,2′–azobis (2,4–dimethylvaleronitrile) (AMVN) and (4–phenyl–1,3–butadienyl)–4–bora–3a, 4a– diaza–*s*–indacene (BODIPY 665/676) as a free radical generator and fluorescent probe, respectively, can be utilized satisfactorily in the determination of lipophilic compounds [69]. Due to this versatility, the range of determined antioxidant substances in meat can be very wide [47]. On the other hand, the ORAC method has certain drawbacks, such as the need of a fluorometer to perform the measurements, the long analysis time, and the high sensitivity to temperature exhibited by the reactions of this test [70] (Table 2), being able to hinder the implementation of this test in laboratories.

#### 2.3.2. Hydroxyl Radical Averting Capacity (HORAC) Assay

The HORAC method was developed by Ou et al. [71] with the objective of detecting the metal chelating capacity of antioxidants in the diet. The basis of this method is identical to ORAC test since an oxidant generator complex and a fluorometric probe are used. The difference that the HORAC test presents compared to the ORAC assay lies in the radical generator complex and in the generated radical itself. Specifically, the HORAC assay uses a Co^2+^–complex to generate hydroxyl radicals (OH^•^), instead of AAPH and ROO^•^ used in ORAC assay, respectively. In this way, a reaction similar to Fenton occurs, a typical reaction of biological systems where OH^•^ is naturally generated from the interaction of H_2_O_2_ and Fe^2+^ [72]. Saving these differences, the HORAC test, in the same way as the ORAC assay employs fluorescein as a probe [73]. Thus, the fluorescein decay curve is monitored when acting against OH^•^ by measuring their intensity at the excitation wavelength of 493 nm and an emission wavelength of 515 nm at least 35 min (at time intervals 0.5 s to 1 min) in a fluorometer [71]. Moreover, in the same way as in the ORAC test, the HORAC results are obtained by calculating the area under the curve that represents the oxidation of the probe over time [73], although the aftermaths in this case are usually expressed as gallic acid equivalents instead of trolox equivalents [71].

Regarding the advantages of this method, there is the use of OH^•^ as an oxidant source since this radical is naturally present in biological systems and can be representative of foods such as meat. Furthermore, this test permit to detect specifically antioxidants that act against the OH^•^ through their ability to chelate metals, thus being an important tool for the study of the preventive antioxidant capacity of foodstuffs [71]. On the other hand, this same specificity means that this method must be combined with other techniques to cover the determination of a greater range of antioxidant compounds. Additionally, its characteristics may mean that it cannot be implemented in all laboratories, since it requires a fluorimeter and uses long reaction times (Table 2).

### 2.4. ET–Based Methods

**Figure 2:**
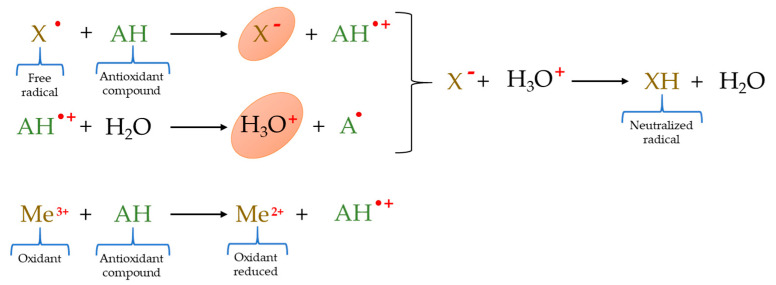
Reactions involved in ET–based methods.

ET–based trials determine the ability of an antioxidant to transfer an electron to reduce any compound (Figure 2) [61]. Specifically, in ET–based assays the probe undergoing reduction with the antioxidant is transformed into a colored, chemiluminescent, or fluorescent substance; or conversely, the initial absorbance, chemiluminescence, or fluorescence of the probe is reduced as a result of the antioxidant reaction [48]. Determinations of the total phenol content (TPC) by Folin–Ciocalteu assay; ferric ion reducing antioxidant power (FRAP) assay; cupric reducing antioxidant capacity (CUPRAC) method; and ferricyanide reducing power belong to ET–based assays [48,58], the TPC test by Folin–Ciocalteu and the FRAP method being the ET–based techniques most used in meat and meat products.

#### 2.4.1. Total Phenol Content (TPC) by Folin–Ciocalteu Assay

The Folin–Ciocalteu assay is a method that allows detecting the antioxidant compounds existing in a food material. Actually, it is not a method for determining antioxidant capacity in the strict sense [58]. However, since the basic mechanism of this technique is a redox–type reaction, Folin–Ciocalteu trial can be considered as another method to determine the antioxidant capacity by electron transfer [61]. Additionally, high contents of phenolic compounds in foods have been associated with high antioxidant capacities [72], confirming the suitability of this test for the determination of this parameter.

**Figure 3:**
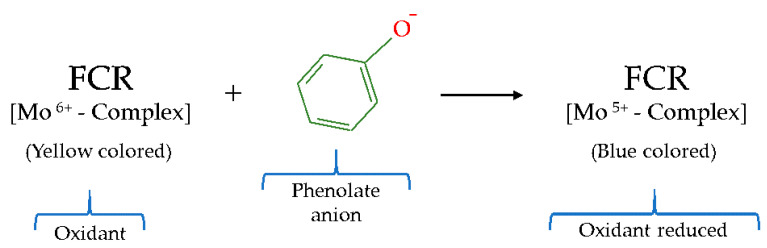
Reduction reaction of Folin–Ciocalteu reagent (FCR).

Initially, the Folin–Ciocalteu test was developed for the detection of proteins by Folin [74], this method being later improved for the determination of phenolic compounds by Singleton and Rossi [75]. The foundation of this test is based on the Folin–Ciocalteu reagent (FCR), which, despite having an unclearly chemical nature, is accepted as containing a mixture of phosphotungstic acid (H_3_PW_12_O_40_) and phosphomolybdic acid (H_3_PMo_12_O_40_) complexes [67,76]. The said complexes are responsible for the redox reaction that occurs between the FCR and the phenolic substances in a basic medium, which generates a blue–colored chromophore with an absorption maximum at a wavelength of 765 nm [67]. This reaction takes place because in a basic medium the phenolic compound undergoes a dissociation of a proton that gives rise to a phenolate anion that can reduce the FCR [73]. It is generally approved that molybdenum (Mo) is responsible for the acceptor of the electron donated by the phenol substance. Thus the phenol compound reduce Mo^6+^ to Mo^5+^ observing an increase in absorbance due to the change in color from an intense yellow (Mo^6+^) to blue (Mo^5+^) color [61] (Figure 3). This absorbance variation can be easily recorded on a UV/Vis spectrophotometer and is directly related to the total phenol content (TPC). In terms of quantification, the TPC is obtained relating the absorbance of the sample with the employ of the standard antioxidant gallic acid, thus expressing the results for TPC as gallic acid equivalents [77,78]. However, sometimes other types of standards are also employed, such as catechin, caffeic acid, chlorogenic acid, or ferulic acid, which can make comparisons between works more difficult [64,79].

The advantages of the Folin–Ciocalteu assay include the simplicity, reproducibility, and robustness of the method [79]. Nevertheless, this technique presents a great drawback since Folin–Ciocalteu is not a specific test for phenolic compounds, but other non–phenolic reducing agents present in the system (particularly sugars, aromatic amines, sulfur dioxide, ascorbic acid, organic acids, Fe^2+^, and other enediols and reductones) may interfere in the phenol quantification and lead to an overestimation of the results [72,80]. Moreover, it is a technique sensitive to temperature, pH, and time, so a correct selection of the operating parameters must be made carefully to maximize an adequate determination [79] (Table 2).

#### 2.4.2. Ferric Ion Reducing Antioxidant Power (FRAP) Assay

**Figure 4:**
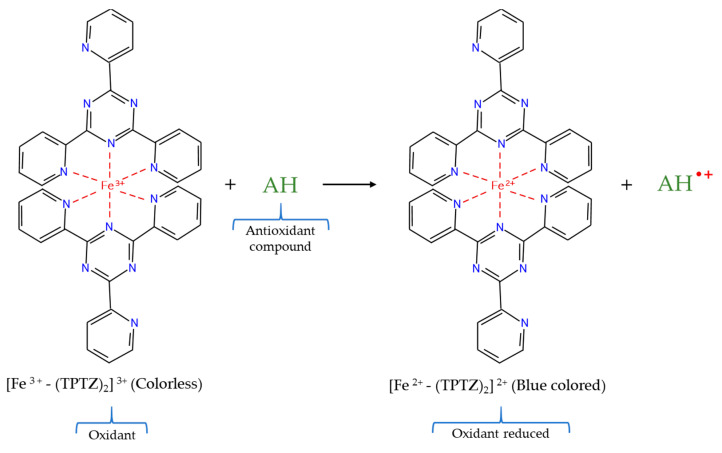
Reduction reaction of FRAP assay.

The FRAP test, initially reported by Benzie and Strain [81] is a typical ET–based assay [58]. This technique measures the ability of antioxidants to reduce ferric ion (Fe^3+^) to ferrous ion (Fe^2+^) [82]. For this, the FRAP assay normally uses the ferric 2,4,6–tripyridyl–*s*–triazine complex [Fe^3+^–(TPTZ)_2_]^3+^, which is an iron salt generated from the mixture of 2,4,6–tripyridyl–*s*–triazine (TPTZ) and FeCl_3_ in acid medium, known as FRAP reagent. In this way, the colorless ferric complex [Fe^3+^–(TPTZ)_2_]^3+^ can be reduced in the presence of antioxidant compounds to the navy blue colored ferrous complex [Fe^2+^–(TPTZ)_2_]^2+^ (Figure 4), which has a maximum of absorbance at a wavelength of 593 nm in acid medium. Thus, the measurement of the increase in absorbance at this wavelength can be carried out quickly (in less than 10 min) and easily through a UV/Vis spectrophotometer which permits monitoring the formation of the [Fe^2+^–(TPTZ)_2_]^2+^ complex [81]. This monitoring of absorbance is linearly correlated with the total reducing capacity of electron–donating antioxidants present in the sample [64], allowing the results to be expressed as Fe^2+^, trolox or ascorbic acid equivalents when Fe^2+^, trolox, and ascorbic acid are used as standards, respectively [58,83,84].

With regard to the advantages of the FRAP method, these are connected to its simplicity, speed, and the lack of need for specialized equipment [58,67], meanwhile its disadvantages are related to the fundamentals of the technique. Thus, because the reactions that occur during FRAP are redox–type, any electron donor substance with a redox potential lower than that of the Fe^3+^/Fe^2+^ pair may contribute to the FRAP value and indicate falsely high FRAP quantities [85,86]. On the contrary, the FRAP assay may also underestimate the antioxidant capacity of certain samples because antioxidants that contain thiol groups (–SH), such as glutathione, cannot be determined through the reactions that occur in the FRAP test [87]. Similarly, antioxidants based on hydrogen transfer such as carotenoids and certain proteins cannot be measured by this method [58,88]. In addition, certain antioxidants, such as caffeic acid, ferulic acid, quercetin, and tannic acid can react slowly with the FRAP reagent, so that the use of reduced reaction times may be insufficient for determine the real antioxidant capacity of samples [89]. On the other hand, the FRAP test is not very representative of a biological system given that it does not use free radicals for the determination of antioxidant capacity. Therefore, the comparisons of antioxidant capacity against different types of free radicals cannot be made. Additionally, a pH of 3.6 is necessary to maintain the solubility of iron cations, which is far from the pH of meat and favor the precipitation of proteins [90]. Furthermore, considering that Fe^2+^ is a pro–oxidant compound, it can generate free radicals such as OH^•^ from hydrogen peroxide [64] which could cause additional oxidation reactions. Finally, the FRAP method can also present interferences due to the fact that certain substances absorb at the wavelength used in this test [91] (Table 2).

### 2.5. Mixed Mode (HAT– and ET–Based) Methods

**Figure 5:**
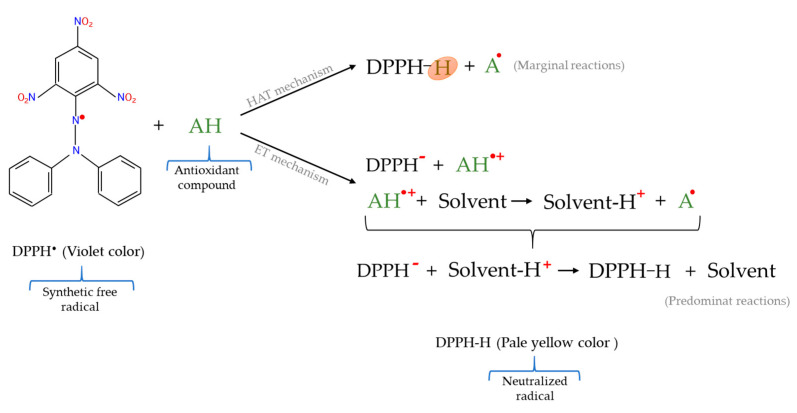
Reactions involved in DPPH^•^ assay.

The mixed mode assays are usually based on the scavenging of a free radical by antioxidants combining the reaction mechanisms of both HAT and ET–based methods and include techniques such as 2,2–diphenyl–1–picrylhydrazyl radical (DPPH^•^) scavenging assay; 2,2′–azinobis–(3–ethylbenzothiazoline–6–sulphonic acid) radical cation (ABTS^•+^) method; and N,N–dimethyl–*p*–phenylenediamine radical (DMPD^•+^) scavenging trial [48]. However, even though the DPPH^•^ method is included in mixed mode assays, it should be considered that the reaction mechanisms that predominate in this technique are ET–based since the abstraction of the hydrogen atom occurs less easily (Figure 5) because this is a slow reaction when accomplished in strong solvents [72,92,93].

On the other hand, among the mixed mode tests cited, the most employed for the determination of the antioxidant capacity in meat and meat products are the DPPH^•^ and ABTS^•+^ assays, which are based on the use of a synthetic and non–biological free radical. Even the use of these techniques in determining the antioxidant capacity of various compounds stand out over the use of techniques HAT– and ET–based methods [94]. 

#### 2.5.1. 2,2–Diphenyl–1–picrylhydrazyl Radical (DPPH^•^) Scavenging Assay

The DPPH^•^ assay was first reported by Blois [95] and is currently a technique judged a standard for the in vitro determination of antioxidants that is extensively employed for the evaluation of free radical scavenging potentials of distinct compounds [96]. This test is characterized by the use of the 2,2–diphenyl–1–picrylhydrazyl free radical, which is a long–lived nitrogen radical specie with an unpaired electron that is delocalized on its entire molecule [97]. The existing delocalization causes the DPPH^•^ radical to have high stability, preventing its dimerization, and giving it an intense violet color typify by an absorption band in organic solution at 515–528 nm [59,98]. Concretely, the DPPH^•^ test is based on the measurement of the reducing capacity of antioxidants against the free radical DPPH^•^. This measurement is generally carried out through the determination of the decrease in the absorbance [61] because when mixing the purple chromogen radical (DPPH^•^) with antioxidant/reducing compounds the color loss occurs with the appearance of the reduced form of DPPH^•^ (DPPH hydrazine), which has a pale yellow color (Figure 5) [94]. In this way, the DPPH^•^ assay simply measures the color loss of DPPH^•^ by monitoring the absorbance decrease in a UV/Vis spectrophotometer at 515–528 nm, until the absorbance remains constant [61,67] since the absorbance diminution depends linearly on the concentration of the antioxidant compounds [83]. Thus, the quantification of antioxidant capacity of a sample can be referred to a standard antioxidant such as trolox, the results being expressed as trolox equivalents [83]. In addition, another usually mode of expression of the antioxidant power used in the DPPH^•^ assay is the IC_50_ value, which represents the antioxidant concentration that provides 50% inhibition of the DPPH^•^ [99]. Therefore, the IC_50_ value is inversely proportional to the radical scavenging activity, and the determined antioxidant capacity.

The advantages presented by the DPPH^•^ technique are high since it is a radical that is commercially available, as well as being a quick and simple method that does not require special pretreatment of the samples [94]. Nonetheless, this procedure has some limitations. For instance, the DPPH^•^ radical is only dissolved in organic media (particularly in alcoholic solutions) and not in aqueous media, which compromises the measurement of hydrophilic antioxidants [100]. On the other hand, DPPH^•^ can interact with other radicals and interferences can also occur due to the fact that certain compounds, such as anthocyanins and carotenoids, absorb in the same wavelength range as DPPH^•^ [93]. Furthermore, the reactions that occur between DPPH^•^ and antioxidant compounds are mainly determined by the steric accessibility because small molecules have better access to the radical site [72]. In this sense, many large molecules can react slowly or even be inert in this test despite having antioxidant capacity. Moreover, DPPH^•^ is a free radical that has no similarity with the peroxyl radicals involved in lipid peroxidation of biological systems [61], which makes it not total representative of samples such as meat. In addition, the DPPH^•^ method is not suitable for emulsions since it reflects the partition of antioxidants at the same time that it can present problems in samples that contain proteins since this molecules precipitate in alcoholic solutions [94] (Table 2).

#### 2.5.2. 2,2′–Azinobis–(3–ethylbenzothiazoline–6–sulphonic acid) Radical Cation (ABTS^•+^) Scavenging Assay

**Figure 6:**
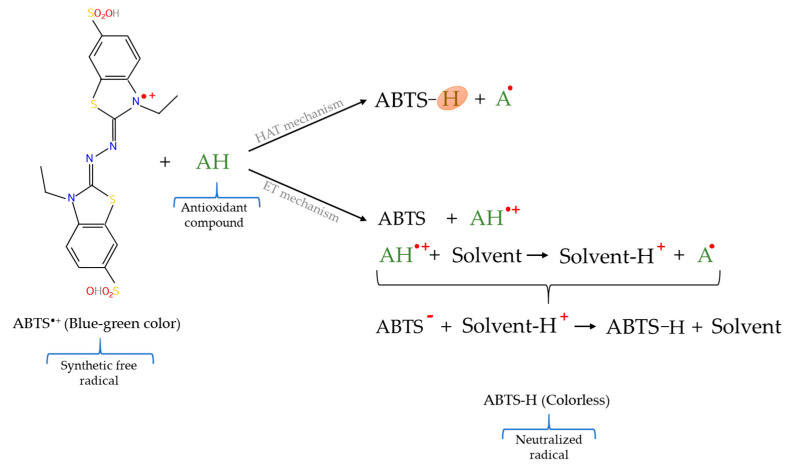
Reactions involved in ABTS^•+^ assay.

The original ABTS^•+^ method, also known as trolox equivalent antioxidant capacity (TEAC), was initially developed by Miller et al. [101]. This technique is based on the generation of a long–life cationic radical, ABTS^•+^, which has a blue–green color with a maximum absorbance at 414, 734, and 815 nm in aqueous medium and at 414, 730, and 873 in ethanolic medium [58]. The ABTS^•+^ is not commercially available, but has to be generated by the emission of an electron from the nitrogen atom that form the molecule of 2,2′–azinobis– (3–ethylbenzothiazoline– 6–sulphonate) (ABTS). This cation formation can be accomplished by oxidation of ABTS via chemical [101,102,103], enzymatic [101,104], or electrochemical reaction [105], being the chemical formation through the use of potassium persulfate, manganese dioxide, or 2,2′–azobis–(2–amidino–propane) dihydrochloride (AAPH), the most widely employed method [94]. In this way, the foundation of ABTS^•+^ test is based on the measurement of the ability of antioxidants to reduce the previously generated cationic radical [45], which leads to a diminution in the coloration and, therefore, a reduction of the absorbance of the sample (Figure 6). Thus, the measurement of antioxidant capacity can be carried out through a UV/Vis spectrophotometer, being the most used wavelength 734 nm because at said wavelength possible interferences from other absorbent components and sample turbidity are minimized [100,106]. In terms of quantification, the ABTS^•+^ values are obtained relating the absorbance diminution of the sample with the use of a standard antioxidant, usually trolox, since the reduction in the absorbance depends linearly on the concentration of the antioxidant substances [83].

Regarding the advantages of the ABTS^•+^ test, the main one is that it is a simple method from the operational point of view, which has allowed it to be a widely used assay for determining antioxidant capacity [61]. In addition, this probe permits the use of a wide pH range [107], and is a rapid test, the reaction time being in most cases less than 30 min in food components [58]. Another very important advantage of this technique is that ABTS^•+^ permits the measurement of both lipophilic and hydrophilic antioxidant compounds since it is soluble in aqueous and organic solvents, and is not affected by ionic strength [108]. On the contrary, the cationic radical ABTS^•+^ is not found in biological systems, representing a non–physiological source of radicals, which can generate results that are not sufficiently representative of foods [88]. In addition, the determination of the antioxidant capacity by ABTS^•+^ can lead to overestimations and underestimations of the antioxidant capacity of the samples, due to the thermodynamics of the reaction and the slowness of the reaction with certain antioxidant compounds, respectively [67] (Table 2).

## 3. Applications of Antioxidant Assays in Meat and Meat Products

In the field of determining the antioxidant capacity of meat and meat–based products, there are limited studies in comparison with other foods such as vegetables, fruits, and spices [47]. However, the use of antioxidant assays has generated special interest, since it allows us to characterize the antioxidant status of this nourishment. In this sense, the determination of the antioxidant capacity has various applications, in fresh meat, meat products, and in protein complexes obtained from these (Table 3). 

### 3.1. Study of the Influence of Animal Diet on the Antioxidant Capacity of Meat

It is widely known that animal nutrition can influence some physicochemical characteristics of meat [35,116,117,118,119,120]. In this regard, the use of antioxidant capacity tests is of particular interest in the characterization of the antioxidant status of meat obtained from differently feedings. Thus, these assays can allow the identification and selection of feedings that favor the enrichment of the meat in antioxidant substances, which could extend the shelf life and improve the quality of meat products. On this matter, the influence of the diet of pigs (as monogastric animals) on the antioxidant status of meat has been one of the most studied via different antioxidant assays. In this regard, Tejerina et al. [112] utilized the ABTS^•+^ and TPC by Folin–Ciocalteu assays to observe the effect of the acorns and grass in the finishing diet of Iberian pigs. Thus, they concluded that the use of this nourishment provided meat from *Longissimus dorsi* and *Serratus ventralis* muscles with a significant better antioxidant status compared to a diet based on complete concentrated diet. Similarly, González et al. [36] reported that acorn–based feeding significant increased the TPC in the adipose tissue of Iberian pigs compared to commercial feed, when the TPC test was carried out by Folin–Ciocalteu test. For their part, Echegaray et al. [35] studied the effect of the inclusion of chestnut on the antioxidant status of different locations of Celta pig (*Longissimus thoracis et lumborum*, *Biceps femoris*, *Psoas major,* and liver) through the FRAP, DPPH^•^, and ABTS^•+^ trials. In this case, the antioxidant capacity assays permitted to conclude that the use of this fruit in the pig finishing diet did not significantly improve the antioxidant capacity of the meat obtained. Similarly, the group of Echegaray et al. [35] observed that the inclusion of chestnut significantly decreased the content of phenolic compounds in meat analyzed through Folin–Ciocalteu test. In addition, due to the combination of techniques for determining the antioxidant capacity employed, they demonstrated that in animal tissue there were compounds other than phenols that exerted antioxidant capacity in meat due to the low correlations found between the TPC and the other tests (FRAP, DPPH^•^, and ABTS^•+^).

Wu et al. [47] used the ORAC method (both for the detection of hydrophilic and lipophilic antioxidants) to evaluate the effects of grazing forage species on *Longissimus dorsi* muscle of Angus–crossbred steers. Specifically, these authors observed how the ORAC test values for hydrophilic compounds were not significantly affected by the finishing diet, while the ORAC values for lipophilic compounds did show significant differences. Thereby, Wu et al. [47] observed that meat obtained from animals fed with alfalfa and pearl millet showed higher lipophilic ORAC values compared to beef meat obtained from steers fed with concentrated feed and native grass. Nevertheless, these authors emphasize that the isolated use of the ORAC test is insufficient to fully detect the influence of diet on the beef antioxidant capacity, once again showing the suitability of combining different methods in the same sample.

On the other hand, Ortuño et al. [37] employed the determination of the antioxidant capacity (using the FRAP, DPPH^•^ and ABTS^•+^ methods) in the *Longissimus thoracis et lumborum* muscle of lambs in order to determine the effect of the inclusion of rosemary diterpenes in the diet of these animals. Thus, after the use of different doses of a dietary rosemary extract, they reported that the antioxidant status of the meat improved compared to control lambs, which were not supplemented. More concretely, they determined that the three antioxidant tests utilized were suitable for the discrimination of antioxidant capacity in lamb meat attributed to rosemary supplementation. However, the DPPH^•^ method proved to be the best test to discriminate the levels of the target diterpenic metabolite (C_19_H_22_O_3_, which is the one with functional properties) in the muscle of lambs, since the DPPH^•^ assay was more dependent on the dose of the extract administered. For all this, Ortuño et al. [37] concluded that rosemary extract could contribute to inhibiting the free radicals generated in oxidized meat. Similarly, Jang et al. [27] observed the effect of a dietary herbal extract (consisting of mulberry leaf, Japanese honeysuckle, and goldthread) in the diet of Cobb broiler chickens via the determination of antioxidant capacity using TPC by Folin–Ciocalteu, DPPH^•^ and ABTS^•+^ methods. They determined that different doses of the herbal extract significantly improved the polyphenol content of chicken breast when compared to chickens fed a control diet. However, these same authors did not obtain a clear trend towards the values of DPPH^•^ and ABTS^•+^ reported during storage under refrigeration for 7 days. Along the same lines, Arshad et al. [113] used the TPC, FRAP and DPPH^•^ assays to determine the effect of a supplementation with *α*–lipoic acid, synthetic *α*–tocopherol, wheat germ oil, and their combinations on the antioxidant status of broiler meat. In this way, they observed that the use of wheat germ oil in combination with α–lipoic acid improved the antioxidant capacity of breast and leg chicken meat measured through the three trials in comparison with a basal diet and with the rest of the supplements (either alone or in combination). These findings showed the greater effectiveness of wheat germ oil (natural α–tocopherol) compared to synthetic α–tocopherol. Additionally, Choi et al. [115] carried out the determination of the antioxidant capacity on breast meat of broilers for the purpose of investigate the influence of the use of different levels of *Hermetia illucens* and *Protaetia brevitarsis seulensis* powders in the feeding of these animals as cheaper animal protein sources. Specifically, Choi et al. [115] employed the DPPH^•^ test through which they observed that supplementation with both insect powders significantly improved antioxidant capacity of breast meat at day 0 compared to a basal diet. However, they also observed that after 7 days of refrigerated storage, the inclusion of powders in the diet did not maintain this improvement in antioxidant capacity of meat. For their part, Kopec et al. [114] studied the effect of dietary histidine supplementation on the antioxidant capacity of turkey breast meat. Specifically, they analyzed through the FRAP, DPPH^•^ and ABTS^•+^ methods the effect of the supplementation with spray dried blood cells rich in histidine and pure histidine on turkey breast meat. This study allowed to conclude that only the supplementation with pure histidine modified the antioxidant capacity of the turkey breast in comparison with a control diet. Furthermore, the authors observed that this increase in antioxidant capacity was only detectable through the DPPH^•^ test, which indicates that in this case said assay is the most suitable for determining antioxidant capacity of turkey meat. Lastly, another example of the importance of determining the antioxidant capacity in meats for the discrimination between diets is found in the work developed by Perna et al. [52]. They studied the effect of the use of a diet enriched in cauliflower powder in rabbits compared to a standard diet. Thus, through the TPC by Folin–Ciocalteu, FRAP, and ABTS^•+^ assays they reported that supplementation with cauliflower powder significantly improved the antioxidant status of *Longissimus lumborum* rabbit muscle. In this way, they determined that this fortification is a valid strategy to produce rabbit meat with better technological and functional qualities.

### 3.2. Study of the Influence of Animal Breed on the Antioxidant Capacity of Meat

In addition to diet, it has been previously observed that breed can affect different parameters of the meat [121,122,123]. The determination of the antioxidant capacity of meat is compelling because it would permit the selection of breeds with good antioxidant status. In this sense, Simonetti et al. [38] employed the study of TPC by Folin–Ciocalteu assay to observe the effect of the pig breed on the content of these potentially beneficial substances in terms of lipid stability. Specifically, they found that Italian autochthonous breed (Suino Nero Lucano) showed a higher content of endogenous total phenols than a modern crossbred pig, both in raw and cooked meat (*Longissimus lumborum* muscle). For their part, Lengkidworraphiphat et al. [39] studied the influence of the chicken genotype on the antioxidant capacity of the breast of three different chickens (Thai indigenous, black–boned, and broiler). Specifically, they combined the techniques of FRAP, DPPH^•^, and ABTS^•+^ to determine which chickens had a better antioxidant status. In this way, they obtained that the Thai indigenous genotype had breast meat with the highest antioxidant capacity compared to the other genotypes. Furthermore, in this study Lengkidworraphiphat et al. [39] concluded that the DPPH^•^ assay was more selective than ABTS^•+^ test in relation to proton donors. Additionally, they observed that the results of the FRAP and DPPH^•^ tests were consistent with each other. Finally, they significantly correlated the carnosine and anserine content of chicken breast with the FRAP and DPPH^•^ tests, demonstrating the suitability of these two methods in the detection of the antioxidant capacity with samples that are rich in these substances.

### 3.3. Study of the Functional Ingredients Addition on the Antioxidant Capacity of Meat Products

The reformulation of meat products is currently on the rise because these foods have been stigmatized in the past two decades due to their relationship with unhealthy products [40,124]. Therefore, the use of functional ingredients, such as natural antioxidants, has acquired an important interest to the detriment of synthetic antioxidants with the aim of offering stability against lipid oxidation [110,125]. In this way, this technological trend has made the analysis of the antioxidant capacity in meat products a compelling parameter when determining the benefits obtained by natural antioxidants. With this purpose, Antonini et al. [40] used the TPC by Folin–Ciocalteu assay and ORAC, DPPH^•^, and ABTS^•+^ tests in cooked beef burgers to which they added chia seeds and/or goji puree in different percentages. These capacity assays allowed them to conclude that the addition of these functional ingredients significantly increased the total phenol content and the antioxidant capacity values (up to 70%) compared to the control burgers. For their part, Duthie et al. [110] employed the HORAC assay and the TPC by Folin–Ciocalteu test to characterize the total antioxidant capacity of cooked patties that contained turkey meat and different vegetable powders. The objective in this case was focused on comparing the results obtained for HORAC with the potential antioxidant substances present in the product (phenols, Vitamin C, and different tocopherols and carotenoids). Thus, they observed that the HORAC values were satisfactorily correlated with the content of free phenols, α–tocopherol, and γ–tocopherol, showing the suitability of this method for determining the antioxidant capacity of foods containing these substances. Other authors utilized the FRAP, DPPH^•^, and ABTS^•+^ methods to determine the antioxidant capacity of rabbit meat products. Specifically, the group of Mancini et al. [41,53] analyzed the effect of the addition of turmeric and ginger powder at different concentrations on the antioxidant status of rabbit meat hamburgers. In this way, they observed that the addition of the turmeric powder to the hamburgers provided an antioxidant status during storage similar to that provided by the additive ascorbic acid [53] and the hamburgers reformulated with ginger powder presented better antioxidant values compared to control hamburgers [41].

On the other hand, the determination of the antioxidant status is also very interesting in meat products that have a replacement of the naturally animal fat by other more unsaturated ones, since the latter are especially sensitive to oxidation [3,126]. In this regard, Carvalho et al. [42] evaluated the effect of guarana seed and pitanga leaf extracts on the antioxidant capacity of different reformulated lamb burgers with a total replacement of animal fat by chia oil. To do this, they used the DPPH^•^ method by which they observed that the addition of vegetable extracts improved the antioxidant capacity until day 6 and 12 of storage under refrigeration when compared with control burgers (without additives) and burgers with the addition of butylated hydroxytoluene (BHT), respectively. Identically, in another study by Carvalho et al. [14] used the DPPH^•^ assay to determine the antioxidant capacity and observe the effect of adding turmeric extract to lamb sausages with a partial substitution of animal fat for tiger nut oil. Through this method, they observed that the addition of turmeric generally improved the antioxidant status of the sausages stored under refrigeration when compared with a negative control (which did not contain additives) and a positive control (which contained sodium erythorbate as antioxidant). Furthermore, de Carvalho et al. [14] observed that turmeric extract exerted its antioxidant capacity at lower doses than the synthetic antioxidant sodium erythorbate. Thus, the DPPH^•^ method permitted them to support the efficacy of turmeric extract in enhancing and maintaining the antioxidant potential of tiger nut–reformulated lamb sausages.

### 3.4. Characterization of the Antioxidant Capacity of Proteins Obtained from Meat

The determination of the antioxidant capacity also is utilized to evaluate the antioxidant status of different protein derivatives obtained from meat and meat by–products [127]. Thus, for example, the HORAC assay was employed by Nishimura et al. [111] to determine the antioxidant capacity of glucose–conjugated chicken myofibrillar proteins. In this case, the HORAC test was used to ensure the obtaining of functional proteins with good antioxidant capacity, which would make it possible to avoid the excessive amounts of antioxidant additives necessary during storage. For their part Borrajo et al. [109] employed the ORAC, FRAP, DPPH^•^, and ABTS^•+^ techniques to determine the antioxidant capacity of porcine liver protein hydrolysates obtained through different conditions (enzymes, times, and filtering). With these four tests, they reported that the ideal conditions for obtaining peptides with antioxidant capacity were the use of Alcalase enzyme with subsequent ultrafiltration through a 30 kDa membrane. However, they displayed differences between ABTS^•+^ and the rest of the methods, since in the case of ABTS^•+^ assay the best hydrolysate was the one that used an ultrafiltrate with a 10 kDa membrane. In this way, the possible differences that may appear between methods and the need to check the antioxidant capacity of a sample via different tests are highlighted. In a similar way, Lee et al. [128] examined the best conditions to obtain hydrolysates through duck skin gelatin with good antioxidant properties. Specifically, these authors observed via the DPPH^•^ method that the use of pepsin reported better results among the nine proteases tested.

## 4. Conclusions

Although research regarding the antioxidant capacity of meat and meat products is currently limited, nowadays there are various analytical methods used to determine antioxidant capacity in theses nourishment. In this sense, the information provided by this review permits to understand the main foundations, advantages, and limitations of the principal antioxidant capacity tests performed in meat and meat products, with the aim of supplying valuable information about these determinations. As a result, this work has revealed the existence of multitude of differences between distinct antioxidant capacity assays in terms of reaction mechanisms, types of substrates, oxidant species and target/probes, reaction conditions, expression of the results, and simplicity. Additionally, this investigation has also frequently evidenced operational differences between the same methods. These occurrences highlight the difficulty in comparing results among different antioxidant capacity assays and between different investigations, even though the method to be compared is the same. Likewise, these facts reveal the need to standardize the analytical procedures for determining the antioxidant capacity, including the previous extraction process, providing valid guidelines that permit the use of these techniques in the routine control of antioxidant capacity measurement of meat and meat products.

On the other hand, the total antioxidant capacity of the meat matrices depends on a multitude of factors; therefore, the combination of different analysis methods is proposed to generate a complete antioxidant profile of these foodstuffs. Specifically, it is recommended to utilize a battery of tests that includes the determination of lipophilic and hydrophilic antioxidants, while allowing the identification of different reaction mechanisms, which take place during the antioxidant reactions. Lastly, this review has also reported various applications of the antioxidant capacity assays in meat and meat products, highlighting the suitability of using these methodologies in different investigations associated to meat and meat products.

## Figures and Tables

**Table 1:** Characteristics of the main methods used in the determination of the antioxidant capacity in meat and meat products.

Reaction Mechanisms	Assay	Oxidizing Agent	Probe	Detection	Monitored Changes
HAT	ORAC	ROO^•^	Fluorescein	Fluorometry	Fluorescence → Non–fluorescence product
	HORAC	OH^•^	Fluorescein	Fluorometry	
ET	Folin–Ciocalteu	Mo^6+^	FCR	Spectrophotometric	Yellow color → Blue color
	FRAP	Fe^3+^	TPTZ	Spectrophotometric	Colorless → Blue color
HAT + ET	DPPH^•^	DPPH^•^ radical	DPPH^•^ radical	Spectrophotometric	Violet color → Pale yellow color
	ABTS^•+^	ABTS^•+^ radical cation	ABTS^•+^ radical cation	Spectrophotometric	Blue–green color → Colorless

HAT: hydrogen atom transfer; ET: electron transfer; ORAC: oxygen radical absorbance capacity; HORAC: hydroxyl radical averting capacity; FRAP: ferric ion reducing antioxidant power; ROO^•^: peroxyl radical; OH^•^: hydroxyl radical; DPPH^•^: 2,2–diphenyl–1–picrylhydrazyl radical; ABTS^•+^: 2,2′–azinobis–(3–ethylbenzothiazoline–6–sulphonic acid) radical cation; FCR: Folin–Ciocalteu reagent; TPTZ: 2,4,6–tripyridyl–s–triazine.

**Table 2:** Principal advantages and disadvantages of the main methods for determining antioxidant capacity in meat and meat products.

Method	Advantages	Disadvantages
ORAC	Versatile technique Useful in complex matrices Representative free radical (ROO^•^)	Specialized equipment necessity (fluorometer) Long reaction times Temperature sensitive
HORAC	Representative free radical (OH^•^)	Specialized equipment necessity (fluorometer) Long reaction times
TPC by Folin–Ciocalteu	Simple method Reproducible technique Robust assay	Detection of possible interferences Temperature sensitive pH sensitive
FRAP	Simple method Quick test No specialized equipment	Substances with lower redox potential than Fe^3+^/Fe^2+^ act as interferences Not quantify antioxidants with –SH groups Not representative conditions of biological systems pH conditions that favor protein precipitation Possible interferences in the measurement of absorbance
DPPH^•^	Simple method Quick test Reactive DPPH^•^ not need previous generation No specialized equipment	Steric impediment of reactions between large molecules and DPPH^•^ Substances with an absorption like DPPH^•^ act as interferences Not appropriate for hydrophilic antioxidants Not suitable for emulsions Causes protein precipitation Not a biological radical
ABTS^•+^	Simple method Quick test Permits working in a wide pH range Useful for hydro– and lipophilic antioxidants No specialized equipment	Requires previous generation of radical Not a biological radical

ORAC: oxygen radical absorbance capacity; HORAC: hydroxyl radical averting capacity; TPC: total phenol content; FRAP: ferric ion reducing antioxidant power; DPPH^•^: 2,2–diphenyl–1–picrylhydrazyl radical; ABTS^•+^: 2,2′–azinobis–(3–ethylbenzothiazoline–6–sulphonic acid radical cation.

**Table 3:** Applications of the principal antioxidant assays employed in meat and meat products.

Assay	Meat Matrix	Purpose of the Antioxidant Capacity Determination	References
ORAC	Angus–crossbred steers meat	Study of the effect of grazing forage species	[47]
	Cooked beef burgers	Research of the addition of chia seeds and/or goji puree	[40]
	Liver protein hydrolysates	Select suitable hydrolysis conditions	[109]
HORAC	Cooked turkey patties	Study of the addition of different vegetable powders	[110]
	Chicken myofibrillar proteins	Ensure the obtaining of functional proteins	[111]
TPC by Folin–Ciocalteu	Iberian pig meat	Study of the influence of the acorns and grass in the pig diet	[112]
	Celta pig meat and liver	Investigation of the effect of chestnut in the pig diet	[35]
	Pig meat	Study of the influence of a local pig breed	[38]
	Cooked turkey patties	Study of the addition of different vegetable powders	[110]
	Cooked beef burgers	Research of the addition of chia seeds and/or goji puree	[40]
	Cobb chicken meat	Contemplation of the effect of a dietary herbal extract	[27]
	Broiler chicken meat	Study of dietary supplementation with natural antioxidants	[113]
	Rabbit meat	Study of the inclusion of a diet enriched in cauliflower powder	[52]
FRAP	Celta pig meat and liver	Investigation of the effect of chestnut in the pig diet	[35]
	Rabbit meat	Study of the inclusion of a diet enriched in cauliflower powder	[52]
	Lamb meat	Study of the effect of dietary rosemary extract	[37]
	Broiler chicken meat	Study of dietary supplementation with natural antioxidants	[113]
	Turkey breast meat	Investigation of dietary supplementation with histidine	[114]
	Chicken meat	Contemplation of the influence of the chicken genotype	[39]
	Rabbit meat hamburgers	Investigation of the effect of turmeric powder addition	[53]
	Rabbit meat hamburgers	Research of the addition of ginger powder	[41]
	Liver protein hydrolysates	Select suitable hydrolysis conditions	[109]
DPPH^•^	Celta pig meat and liver	Investigation of the effect of chestnut in the pig diet	[35]
	Lamb meat	Study of the effect of dietary rosemary extract	[37]
	Cobb chicken meat	Contemplation of the effect of a dietary herbal extract	[27]
	Broiler chicken meat	Study of dietary supplementation with natural antioxidants	[113]
	Arbor Acres chicken meat	Research of the employ of insects as protein sources in chicken diet	[115]
	Turkey breast meat	Investigation of dietary supplementation with histidine	[114]
	Chicken meat	Contemplation of the influence of the chicken genotype	[39]
	Cooked beef burgers	Research of the addition of chia seeds and/or goji puree	[40]
	Rabbit meat hamburgers	Investigation of the effect of turmeric powder addition	[53]
	Rabbit meat hamburgers	Research of the addition of ginger powder	[41]
	Reformulated lamb hamburgers	Study of guarana seed and pitanga leaf extracts addition	[42]
	Reformulated lamb sausages	Investigation of the addition of turmeric extract	[14]
	Liver protein hydrolysates	Select suitable hydrolysis conditions	[109]
ABTS^•+^	Iberian pig meat	Study of the influence of the acorns and grass in the pig diet	[112]
	Celta pig meat and liver	Investigation of the effect of chestnut in the pig diet	[35]
	Lamb meat	Study of the effect of dietary rosemary extract	[37]
	Cobb chicken meat	Contemplation of the effect of a dietary herbal extract	[27]
	Turkey breast meat	Investigation of dietary supplementation with histidine	[114]
	Rabbit meat	Study of the inclusion of a diet enriched in cauliflower powder	[52]
	Chicken meat	Contemplation of the influence of the chicken genotype	[39]
	Cooked beef burgers	Research of the addition of chia seeds and/or goji puree	[40]
	Rabbit meat hamburgers	Investigation of the effect of turmeric powder addition	[53]
	Rabbit meat hamburgers	Research of the addition of ginger powder	[41]
	Liver protein hydrolysates	Select the suitable hydrolysis conditions	[109]

ORAC: oxygen radical absorbance capacity; HORAC: hydroxyl radical averting capacity; TPC: total phenol content; FRAP: ferric ion reducing antioxidant power; DPPH^•^: 2,2–diphenyl–1–picrylhydrazyl radical; ABTS^•+^: 2,2′–azinobis–(3–ethylbenzothiazoline–6–sulphonic acid.
